# High-Performance Metal/Carbide Composites with Far-From-Equilibrium Compositions and Controlled Microstructures

**DOI:** 10.1038/srep35523

**Published:** 2016-10-18

**Authors:** Liangfa Hu, Morgan O’Neil, Veysel Erturun, Rogelio Benitez, Gwénaëlle Proust, Ibrahim Karaman, Miladin Radovic

**Affiliations:** 1 Department of Materials Science and Engineering, Texas A&M University, College Station, TX 77843, USA; 2 Department of Mechanical Engineering, Texas A&M University, College Station, TX 77843, USA; 3 Department of Airframes and Powerplants, Erciyes University, Kayseri 38039, Turkey; 4 School of Civil Engineering, University of Sydney, NSW 2006, Australia

## Abstract

The prospect of extending existing metal-ceramic composites to those with the compositions that are far from thermodynamic equilibrium is examined. A current and pressure-assisted, rapid infiltration is proposed to fabricate composites, consisting of reactive metallic and ceramic phases with controlled microstructure and tunable properties. An aluminum (Al) alloy/Ti_2_AlC composite is selected as an example of the far-from-equilibrium systems to fabricate, because Ti_2_AlC exists only in a narrow region of the Ti-Al-C phase diagram and readily reacts with Al. This kind of reactive systems challenges conventional methods for successfully processing corresponding metal-ceramic composites. Al alloy/Ti_2_AlC composites with controlled microstructures, various volume ratios of constituents (40/60 and 27/73) and metallic phase sizes (42–83 μm, 77–276 μm, and 167–545 μm), are obtained using the Ti_2_AlC foams with different pore structures as preforms for molten metal (Al alloy) infiltration. The resulting composites are lightweight and display exceptional mechanical properties at both ambient and elevated temperatures. These structures achieve a compressive strength that is 10 times higher than the yield strength of the corresponding peak-aged Al alloy at ambient temperature and 14 times higher at 400 °C. Possible strengthening mechanisms are described, and further strategies for improving properties of those composites are proposed.

Unusual, often unique, properties of MAX phases that are uncommon for most of the typical ceramics or metals^1^ have fueled recent research on the development of metal/MAX phase composites for a variety of applications. These properties include high-temperature stability, thermal shock resistance, damage tolerance^2^, good transport properties, and machinability^1^. The MAX phases have layered, nano-laminated structures and share a common chemical formula, M_n+1_AX_n_ (where n = 1, 2, or 3, where M is an early transition metal, A is an A-group element mostly from groups 13–16, and X is carbon and/or nitrogen).

Since Ti_3_SiC_2_ (one of the most common MAX phase materials) was proposed for the first time as a reinforcement phase for copper (Cu) in an electro-friction composite^3^, a number of MAX phases have been used to increase the mechanical strength of Cu or aluminum (Al) without reducing thermal and electrical conductivity of both metals^4^,^5^. An addition of silver (Ag) in Ta_2_AlC and Cr_2_AlC has been shown to improve the tribological performance of the ternary carbides; the resulting composites were proposed as new solid lubricants against Ni-based super-alloys and alumina^6^. The yield strength of a Ti_3_AlC_2_/Al composite was found to be twice that of Al^7^. Previous investigations have shown remarkably higher mechanical energy dissipation in NiTi/Ti_3_SiC_2_^8^,^9^,^10^ and Mg/Ti_3_SiC_2_^11^,^12^ composites than that in their pure constituents. All composites mentioned above were processed by powder co-sintering method.

A challenging issue of the powder co-sintering method is the reaction between metals and the MAX phases. For example, the Ti_3_AlC_2_/Al composites were sintered at a much lower temperature (550 °C) than the usual sintering temperature of Ti_3_AlC_2_ to avoid substantial and harmful reactions, and thus Ti_3_AlC_2_ particles were barely sintered in those composites^7^. The temperature choice in the powder co-sintering method is dictated by the melting point of the metals, which is normally well below typical sintering temperatures of MAX phases, but high enough to trigger intensive reactions between the constituents. One way to process metal/MAX phase composites with controlled interfacial reactions is to infiltrate molten metals into MAX phase foams. In fact, a pressureless infiltration technique has been used to process Ti_2_AlC/Mg composites that exhibit higher strength and mechanical energy dissipation than other Mg composites^11^,^12^. However, the pressureless infiltration is not an easy task because of the poor wettability of MAX phase foams with molten metals. The poor wettability slows the infiltration and sometimes makes it impossible. It can also yield a weak bonding between metals and ceramics, resulting in inferior mechanical properties of the composites^13^. This problem can be overcome using pressure infiltration to force molten metals into ceramic foams. However, in many cases the ceramic-metal reaction is so fast that even during pressure infiltration new phases would form, especially at the interface between the MAX phase and the metallic alloy. The new phases not only block pores and thus prevent further infiltration, but also degrade the properties of the constituents in the fabricated composites. Therefore, reducing the chemical reaction is the most challenging issue for fabricating metal/MAX phase composites with far-from-equilibrium compositions. In the present work, an Al alloy/Ti_2_AlC composite is selected as an example of far-from-equilibrium systems to fabricate, because Ti_2_AlC exists only in a narrow region of the Ti-Al-C phase diagram and readily reacts with molten Al at temperatures above 660 °C.

Al is a natural choice to be used in ceramic-metal composites for aerospace and transportation applications, where weight saving is critical. Particularly, the development of new generation aircrafts calls for high-performance Al alloys, especially at elevated temperatures. An approach to obtain improved high-temperature properties is to combine Al alloys with ceramics, such as Al_2_O_3_^14^,^15^,^16^, B_4_C^17^,^18^,^19^, and SiC^20^,^21^,^22^,^23^,^24^. However, MAX phases have not been explored for Al-based composites until two recent studies on Ti_3_AlC_2_/Al^7^ and Al alloy/Ti_2_AlC composites^25^.

The introduction of MAX phases in Al composites brings several additional advantages, which could not otherwise be obtained using traditional ceramics, *e.g.* Al_2_O_3_, B_4_C, or SiC. First, a typical MAX phase, like Ti_3_SiC_2_, has a higher fracture toughness (~7 MPa·m^1/2^) than Al_2_O_3_ (~4 MPa·m^1/2^), B_4_C (~3.7 MPa·m^1/2^), and SiC (~4.6 MPa·m^1/2^) that potentially can reduce the sensitivity of composites to brittle fracture. Secondly, unlike traditional ceramics, MAX phases have good transport properties that originate from the atomic bonding with mixed covalent, ionic, and metallic characters. Good transport properties of MAX phases would retain the functional properties of Al, namely good thermal and electrical conductivities. Third, MAX phases can be compressed to stresses as high as 1 GPa and fully recover their original, undeformed shapes upon the removal of the stress while dissipating 25% of the mechanical energy. The addition of MAX phases in Al can introduce mechanical energy dissipation, which is unique when compared to traditional ceramic-Al composites. Lastly, unlike traditional ceramics, MAX phases are machinable even at room temperature, which can significantly reduce the manufacturing cost of the composite.

It was shown in our previous work that a current and pressure-assisted, rapid infiltration is a viable method to produce Al alloy/MAX phase composites^25^. In the present work, the combination of rapid infiltration and MAX phase foams with tailored structures is used to control the microstructure in Al alloy/MAX phase composites. In addition, we characterize the microstructures and perform micro-tomography on the resulting composites to find out the reaction mechanisms during processing, and finally tailor the compressive strength of the composites by controlling their microstructures. We also discuss the possibility of adapting this method in building complex hierarchical structures to mimic natural composites.

## Results and Discussion

### Microstructural, Compositional, and Phase Analyses

**Figure 1:**
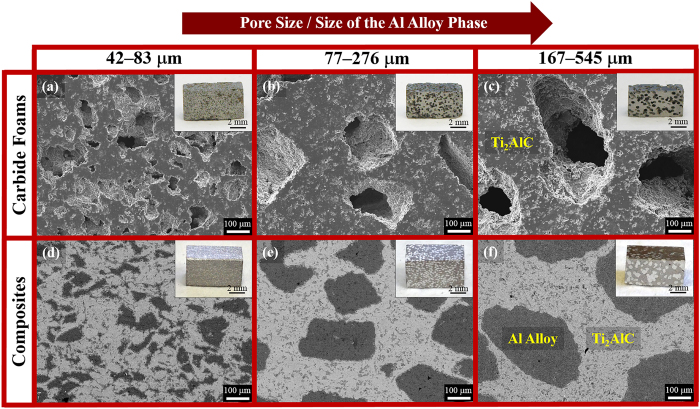
SEM images of Ti_2_AlC foams with various pore sizes fabricated using NaCl particles as a pore former. The pore sizes are in the range of (**a**) 42–83 μm, (**b**) 77–276 μm, and (**c**) 167–545 μm. All three foams were fabricated using the same volume percent (40 vol.%) of the NaCl particles. The infiltration of these three foams with the Al 6061 alloy resulted in Al alloy/Ti_2_AlC composites with various sizes of the Al alloy phase, namely (**d**) 42–83 μm, (**e**) 77–276 μm, and (**f**) 167–545 μm. The sizes of the Al alloy phase are pore sizes in the foams before infiltration. Insets show photographs of both the foams and the composites after electrical discharge machining to dimensions of 3.5 mm × 3.5 mm × 7 mm.

Figure 1a–c show SEM images of the Ti_2_AlC foams with different pore sizes, *i.e.* 42–83 μm, 77–276 μm, and 167–545 μm, respectively, fabricated using NaCl particles as pore formers^26^,^27^. All three foams were fabricated using the same volume percent (40 vol.%) of NaCl particles and have comparable overall porosities of 40.8, 41.6 and 39.9 vol.% after pressureless sintering. The infiltration of these foams with Al 6061 alloy using procedures described elsewhere^25^ resulted in Al alloy/Ti_2_AlC composites with various sizes of the Al alloy phase, as shown in Fig. 1d–f. The size of the Al alloy phase is identical to the pore size in the Ti_2_AlC foams before infiltration. To illustrate and compare the macroscopic structures, insets in Fig. 1 show photographs of both the foams and the composites. Despite the short infiltration time of approximately 30 seconds, the porosity measurement results (see section “Mechanical Properties under Compression”) indicate that more than 94% of the open pores in all foams were infiltrated with the molten Al alloy. In addition, a small amount of reaction could be observed at interfaces between the two major phases on SEM images in Fig. 1. Further analysis of the minor interfacial reaction is presented below.

**Figure 2:**
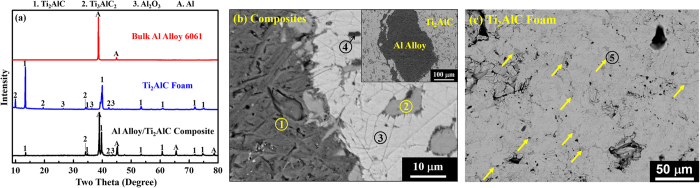
(**a**) XRD results of the Al alloy/Ti_2_AlC composite and its initial constituents, namely Al alloy and Ti_2_AlC. The identification of the phases was carried out using the Inorganic Crystal Structure Database (ICSD) collection code 165460 for Ti_2_AlC, 153266 for Ti_3_AlC_2_, 10425 for Al_2_O_3_, and 43423 for Al. (**b**) A backscattered SEM image of the composite showing Al alloy (1, dark grey), titanium aluminide (2, light grey), Ti_2_AlC (3, light), and Al_2_O_3_ (4, dark). Inset shows a backscattered SEM image at a low magnification. The composition of phases 1, 2, 3, 4, and 5 are determined according to the EDS results (Table 1) and EBSD analysis (Fig. 3). (**c**) A backscattered SEM image of the Ti_2_AlC foam with arrows pointing out to TiAl_2_ impurities (stoichiometry determined by EDS).

**Figure 3:**
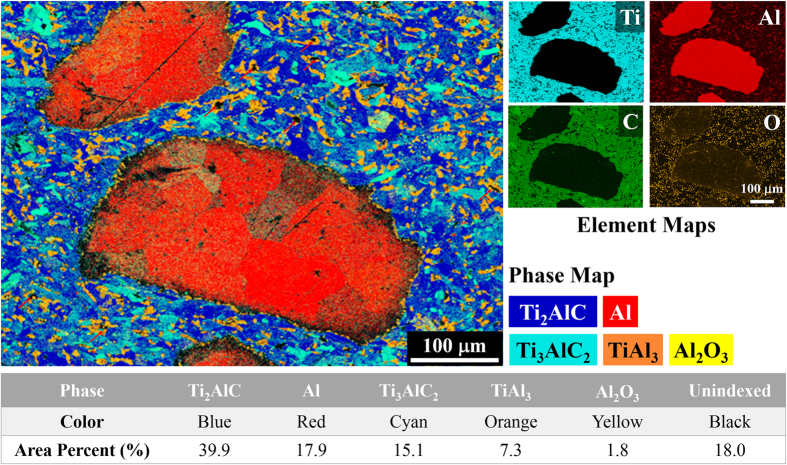
A phase map and four element maps, namely Ti, Al, C, and O, of the Al alloy/Ti_2_AlC (volume ratio 40/60) composite with the size of the Al alloy phase ranging from 167 to 54 μm. Both identified and unindexed phases are color coded in the phase map; 82.0% of all phases were identified, and 18.0% were not identified. The identified phases include Ti_2_AlC, Ti_3_AlC_2_, Al, TiAl_3_, and Al_2_O_3_; the area percent of each phase is listed in the table. Red arrows point to some of the unidexed phases.

Figure 2a shows X-ray diffraction (XRD) results of the composites in comparison with the starting materials (*i.e.* Al alloy and Ti_2_AlC). In addition to the major phase (Ti_2_AlC), the commercial MAXthal211 (Sandvik Materials Technology, Hallstahammar, Sweden) powder used in the present study contains Ti_3_AlC_2_ and a small amount of Al_2_O_3_ (Fig. 2a). Ti_2_AlC, Ti_3_AlC_2_ and Al were three major phases in the processed composites, together with a small amount of Al_2_O_3_. All phases identified in the composites were also found in the starting materials, and no new phases were detected in the XRD. However, more detailed compositional and phase analysis of the composites by energy dispersive spectrometry (EDS) (Fig. 2b,c) and electron backscatter diffraction (EBSD) (Fig. 3) clearly showed the presence of an intermetallic (TiAl_3_) phase, whose amount was most likely too small to be detected by using XRD.

Figure 2b shows typical backscattered SEM images of the Al alloy/Ti_2_AlC composite at different magnifications, while the EDS results in Table 1 include the concentration of individual elements, Ti/Al ratio, and Al/O ratio in each spot indicated in Fig. 2b, as well as a summary of the phases detected in the EBSD analysis in Fig. 3. Note that carbon is not shown in the EDS results, because EDS cannot provide carbon content with a high accuracy. The Ti/Al ratios in spots 2 and 3 are 0.35 and 1.96 (EDS results), respectively, which are in good agreement with the Ti/Al ratios in TiAl_3_ and Ti_2_AlC, respectively. The presence of those two phases was also confirmed by EBSD, Fig. 3. Similarly, the Al/O ratio of 0.69 in spot 4 suggests Al_2_O_3_ phase, whose presence was also confirmed by the EBSD results (Fig. 3). While the intermetallic phase was most likely formed by the reaction between molten Al alloy and MAXthal211, as it is discussed in more detail below, Al_2_O_3_ has three possible sources. These sources include raw powder (Fig. 2c), possible surface oxidation of Al alloy during processing, and possible reaction between Al and an oxide layer or oxide adsorbates on the surface of Ti_2_AlC preform.

As a further confirmation of the phase composition of the composites, Fig. 3 shows a phase map and four element maps (Ti, Al, C, and O) of the composites with the sizes of the Al alloy phase ranging from 167 to 545 μm. Both identified and unindexed phases are color coded in the phase map. As listed in Fig. 3, 82.0% of all phases were identified, and 18.0% were not identified by EBSD. Note that out of the 18.0% unindexed phases, approximately 6.0% is residual porosity in the composites (see section “Mechanical Properties under Compression”). The identified phases include Ti_2_AlC, Ti_3_AlC_2_, Al, TiAl_3_, and Al_2_O_3_. The area percent of each phase is listed in Fig. 3. Note that Al_2_O_3_ is present in both ceramic and metallic phase regions, suggesting that the Al_2_O_3_ in the ceramic phase region most likely comes from the raw ceramic powder (Fig. 2), whereas the Al_2_O_3_ in the metallic phase region should result from surface oxidation of the Al alloy or Al powders used in this study. A significant amount of TiAl_3_, *i.e.* 7.3% (Fig. 3), was identified either at the interfacial region or in the ceramic phase regions. The TiAl_3_ in the interfacial region forms a thin “ring” surrounding the Al alloy, separating it from the ceramic phases. A comparison between the phase map and SEM images of the Ti_2_AlC foam (Fig. 1) suggests that the locations of the TiAl_3_ at the ceramic phase region could formerly be the small pores in the ceramic walls, where molten Al alloy could reach and react with the TiAl_2_ found in the Ti_2_AlC foam (Fig. 2c).

The unindexed, black regions in Fig. 3 have three possible sources. First, the black dots with sizes of approximately 10 μm in the Ti_2_AlC region are most likely pores not infiltrated with the Al alloy. Second, the black area, especially around interfaces, were unidentified, because the area was in electron shadow during EBSD data collection due to a slight height difference between the two phases as a result of their uneven polishing of harder ceramic and softer Al phases. Third, the black, straight lines with lengths of 50–150 μm are scratches, which were introduced during polishing.

A common reaction phase, titanium aluminide, was found in both the composites presented here and the previously reported Ti_3_AlC_2_/Al composites^7^, as it is expected from the Ti-Al-C phase diagram^28^. However, the composites presented here have significantly less titanium aluminide than previously reported^7^. Thus, the short processing time plays an important role in reducing reactions, and the short time associated with the rapid infiltration in the present study becomes particularly prominent in the case of reactive ceramic-metal systems. To that end, two possible mechanisms could explain the formation of TiAl_3_ and are provided in the Supplementary Information section.

### Mechanical Properties under Compression

It has been demonstrated up to this point that the rapid infiltration allows the processing of new composites with customizable microstructure by controlling the size and volume fraction of the metal and ceramic constituents of the Al alloy/Ti_2_AlC composites. The section below points out that the customized structures could be utilized to tailor mechanical properties and that the rapid infiltration can result in superior mechanical properties of the composites in comparison with their pure constituents.

#### Effects of the Volume Fraction and Size of the Al Alloy phase on Mechanical Properties

**Figure 4:**
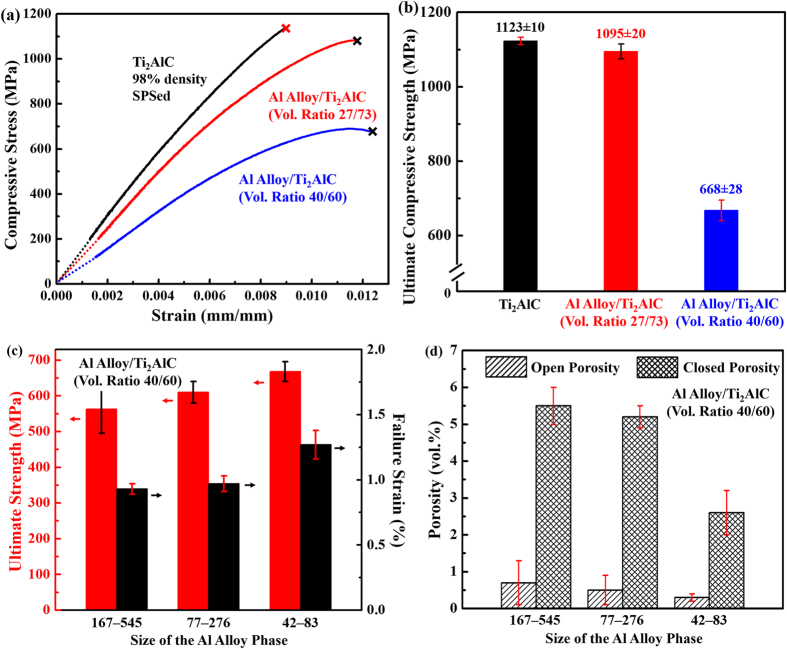
(**a**) Typical compressive stress-strain curves and (**b**) ultimate compressive strength of Ti_2_AlC (98% relative density, spark plasma sintered (SPSed)), the Al alloy/Ti_2_AlC (volume ratio 27/73) composites, and the Al alloy/Ti_2_AlC (volume ratio 40/60) composites. (**c**) Ultimate compressive strength and failure strain of the composites with various sizes of the Al alloy phase. (**d**) Open and closed porosity of the composites as a function of the size of the Al alloy phase. The size of the Al alloy phase is the pore size of the Ti_2_AlC foams before infiltration.

Figure 4a shows the typical compressive stress-strain curves at room temperature for Al alloy/Ti_2_AlC (volume ratios 40/60 and 27/73) and Ti_2_AlC, while Fig. 4b summarises their room temperature compressive strengths. Figure 4c presents both the ultimate compressive strength and failure strain of the Al alloy/Ti_2_AlC composites with 40 vol% Al alloy phase of different size (42–83 μm, 77–276 μm, and 167–545 μm). Both the strength and failure strain of the composites decrease with the size of the Al alloy phase. For example, the compressive strength and failure strain of the composites with the sizes of the Al alloy phase in the range of 42–83 μm are approximately 20% and 35%, respectively, higher than those of the composites with the sizes of the Al alloy phase in the range of 167–545 μm. The differences in the compressive strengths and failure strains lie in the differences in porosity and phase boundary area that are responsible for arresting crack propagations, each of which is illustrated or explained below.

Phase boundary area is responsible for arresting crack propagations (see Supplementary Information), and the difference in the phase boundary area relates to the differences in the compressive strength and failure strain. Given a fixed volume percent of interpenetrating phase, the phase boundary area increases with decreasing size of the Al alloy phase. With the largest phase boundary area, the composite with the finest size of the Al alloy phase has the most effective crack arresting and deflection capabilities and thus exhibits the highest compressive strength and failure strain.

#### Mechanical Properties at Both Room and Elevated Temperatures

**Figure 5:**
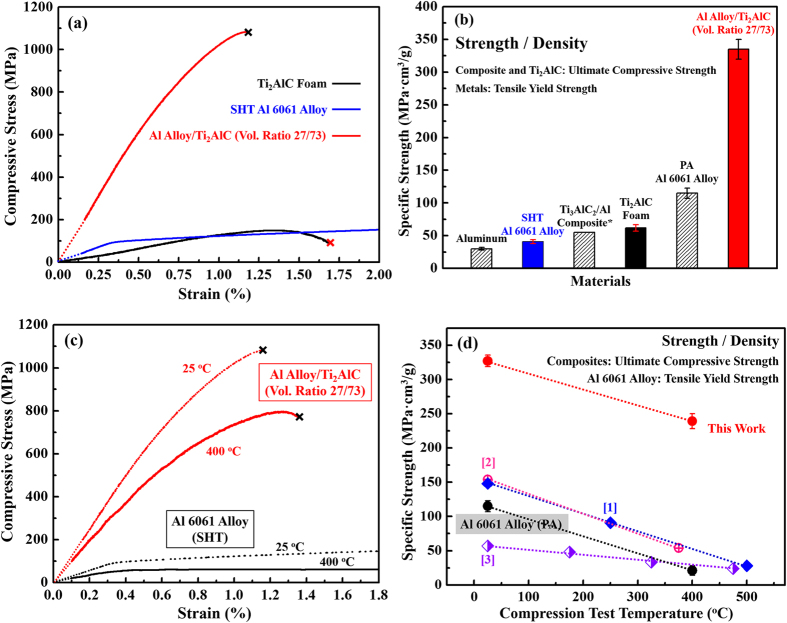
(**a**) Typical stress-strain curves of the Al alloy/Ti_2_AlC (volume ratio 27/73) composites, solution heat treated (SHT) Al 6061 alloy, and Ti_2_AlC foams. (**b**) Specific strength of the Al alloy/Ti_2_AlC (volume ratio 27/73) composites, peak aged (PA) Al 6061 alloy, and Ti_2_AlC foams, previously reported Ti_3_AlC_2_/Al composites^7^, SHT Al 6061 alloy, and pure Al. (**c**) Typical stress-strain curves of the Al alloy/Ti_2_AlC (volume ratio 27/73) composites and SHT Al 6061 alloy at 25 °C and 400 °C. (**d**) Specific strength as a function of test temperature for the Al alloy/Ti_2_AlC (volume ratio 27/73) composites from the present study (red), previously reported composites (i.e. Ti_3_AlC_2_/Al^7^ (blue [1]), B_4_C/Al^17^ (pink [2]), and Al_2_O_3_/Al^15^ (purple [3]), and PA Al 6061 alloy (black).

Figure 5a shows typical compressive stress-strain curves of the Al alloy/Ti_2_AlC composites, solution heat treated (SHT) Al 6061 alloy, and Ti_2_AlC foam. At room temperature, the Al alloy in the composite should have mechanical properties comparable with SHT Al 6061 alloy due to the rapid high-temperature processing. The ultimate compressive strength of the composite is 1095 MPa, which is about 7 times that of the Ti_2_AlC foams (150 MPa) and about 10 times the yield strength of the SHT Al 6061alloy (110 MPa^29^). Figure 5b shows that the specific strength, *i.e.* compressive/yield strength divided by density, of the composite is 335 MPa·cm^3^/g, which is about 5 times that of the Ti_2_AlC foam (62 MPa·cm^3^/g), 3 times that of the peak aged (PA) Al 6061 alloy (115 MPa·cm^3^/g^29^), 6 times that of previously reported value of the Ti_3_AlC_2_/Al composite (55 MPa·cm^3^/g^7^), and 5 times the yield strength of the SHT Al 6061 alloy (41 MPa·cm^3^/g). The importance of the later cannot be overemphasized, especially for applications in which lightweight materials that can carry large loads, such as aerospace and transportation, are essential.

Figure 5c shows typical compressive stress-strain curves of the composites and PA Al 6061 alloy at 25 °C and at 400 °C. Note that Al alloy creeps at 400 °C, which is approximately 0.7 of its melting point. The ultimate compressive strength of the composites at 400 °C (800 MPa) is more than one order of magnitude the yield strength of Al 6061 alloy at 400 °C (58 MPa). Although the strength of the composites is higher than that of PA Al 6061 alloy at both room and elevated temperatures, the failure strain of the composites is smaller than that of PA Al 6061 alloy. In other words, the ceramic phase strengthens Al alloy at the expense of its ductility.

#### Thermal Stability

**Figure 6:**
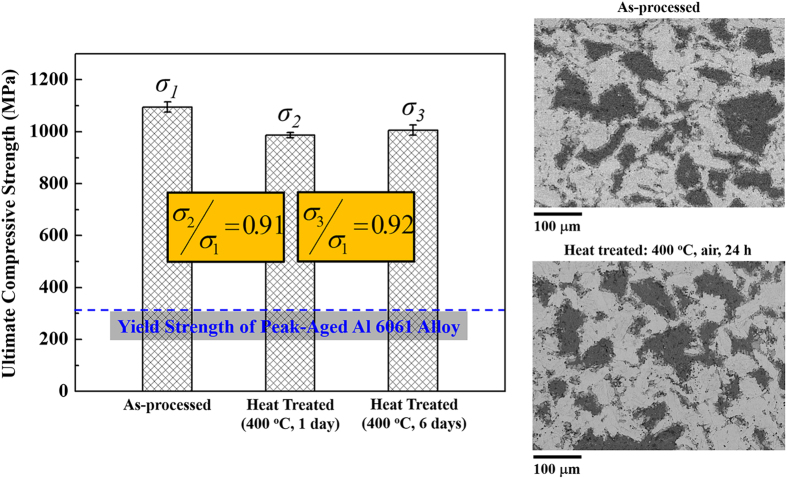
Comparison of ultimate strength and backscattered SEM images (dark: Al alloy, grey: TiAl_3_, and light: Ti_2_AlC) between as-processed and heat treated Al alloy/Ti_2_AlC (volume ratio 27/73) composites. The heat treatments were at 400 °C (0.7 of the melting point of Al) for 1 day and 6 days. The blue, dash line in the plot indicates the yield strength of peak-age Al 6061 alloy.

Since the Al alloy/Ti_2_AlC is a far-from-equilibrium system, thermal stability is equally important as mechanical strength at elevated temperatures. Figure 6 shows, side by side, the ultimate compressive strength and backscattered SEM images of the as-processed and heat treated Al alloy/Ti_2_AlC (volume ratio 27/73) composites. After heat treatments at 400 °C (0.7 of the melting point of Al) for one day and six days, the composites retained 91% and 92% of its strength, respectively, suggesting a sustainable mechanical performance against elevated temperatures. The backscattered SEM images of the composites before and after the heat treatment suggest little growth of the reaction phase and no evidence of interface de-bonding or cracks at the micrometer scale, which is in good agreement with the fact that the composites were able to retain more than 90% of its strength after the heat treatment.

The difference of the compressive strength before and after the heat treatment could come from several changes. First, the heat treatment could relax the residual stresses introduced during the rapid cooling, leading to different initial stress states between as-processed and heat treated composites for both metallic and ceramic phases upon loading. Second, the heat treatment could change the small misorientation within Al alloy grains. Third, the heat treatment could dissolve precipitates (if any) in the Al alloy. The last two possible changes would lower the strength of Al alloy in comparison with the Al alloy in the as-processed composites.

## Conclusions

The current and pressure-assisted, rapid infiltration was used for producing interpenetrating Al alloy/Ti_2_AlC composites with controlled volume percent and size of the Al alloy phase, which were utilized to tailor compressive mechanical properties of the composites. The major findings are summarized as follows.

1. More than 97% of the open porosity in the ceramic preform was infiltrated with molten metal even after a short processing time of approximately 30 seconds. The results suggest that the rapid infiltration offers an efficient route for producing interpenetrating ceramic-metal composites with customizable structures by controlling the structure of the ceramic preform.
2. Little reaction was observed in the Al alloy/Ti_2_AlC system, suggesting that the rapid infiltration could be used to process far-from-equilibrium composite systems, which could not otherwise be obtained using conventional methods.
3. This method can result in composites with superior properties in comparison with those of its constituents. The specific compressive strength of the Al alloy/Ti_2_AlC composites is about 10 times and 14 times higher than the specific yield strength of peak-aged Al alloy at room temperature and at 400 °C, respectively.
4. Despite the far-from-equilibrium composition, the Al alloy/Ti_2_AlC composites are thermally stable up to 400 °C (0.7 of the melting point of Al). The Al alloy/Ti_2_AlC (volume ratio 27/73) composites retained more than 90% of its strength after a heat treatment at 400 °C for six days.

## Methods

### Materials

Ti_2_AlC powders (MAXthal 211, Sandvik Heating Technology AB, Sörkvarnsvägen, Sweden) with the particle size in the 45–90 μm range, and NaCl powders (Sigma-Aldrich, St. Louis, MO, USA) with a variety of particle sizes, *i.e.* 45–90 μm, 180–250 μm, and 355–500 μm, were used to process Ti_2_AlC foams following the procedures described elsewhere^26^,^27^. The preparation of the foams involves three steps: (i) cold pressing a NaCl-Ti_2_AlC powder mixture with 20/80 or 40/60 volume ratio; (ii) dissolving the NaCl in distilled water, and (iii) pressureless sintering of the Ti_2_AlC under flowing argon at 1400 °C for 4 hours^27^. Note that powder mixtures with other volume ratios were also processed to yield Ti_2_AlC foams with a variety of porosity (ranging from 10–71 vol.%) and pore size (ranging from 42–545 μm)^26^,^27^. Only two ratios (20/80 and 40/60) were selected in the present study for proof-of-concept work of rapid infiltration with these Ti_2_AlC foams. Further investigations could be carried out to elaborate the effects of porosity and pore size of the ceramic foams on the infiltration process and properties of the resulting composites.

Pore size of the foams was determined by measuring the size of approximately 50 pores in SEM images using the line intercept method, as specified in ASTM E112–13^30^. Four SEM images from randomly selected locations on each sample were used to measure the pore size. Al 6061 alloy discs (McMaster-Carr, GA) with a diameter of 20 mm and a thickness of 4 mm were used for the infiltration process.

### Composite Sample Preparation

A disc (20 mm in diameter and 4 mm in thickness) of Ti_2_AlC foam was “sandwiched” in between two Al alloy discs and placed in a graphite die, followed by infiltration at 750 °C under 5 MPa uniaxial pressure for 1 minute. The “sandwich” set-up enables more uniform infiltration of molten metal. The infiltration was carried out in a spark plasma sintering system (SPS 25-10, GT Advanced Technologies, CA). A direct current was applied from 0 to 1250 A in 4 min and stabilized at 860 A for 1 min at 750 °C; the pulse cycle was 10 ms on and 10 ms off. The chamber was evacuated and held at 10^−6^ torr for 10 minutes before heating. The heating rate was 200 °C/minute. It takes less than 10 minutes for a complete infiltration process including heating/melting, soaking, and cooling/solidification. Graphite foils were applied between samples and graphite die before infiltration. The temperature was calibrated and measured using procedures described elsewhere^8^.

### Characterization

The density and porosity (both open and closed) were determined by alcohol immersion method based on Archimedes’ principle, as specified in ASTM C20-00^31^. The theoretical density values of 4.11 (g/cm^3^)^32^ and 2.70 (g/cm^3^)^32^ for Ti_2_AlC and Al, respectively, were used to calculate the theoretical density of composites using the rule of mixture. It was assumed that the effect of new phases formed by chemical reactions on the theoretical density is negligible. The relative density equals the measured value divided by the ROM value, *i.e.* 3.55 g/cm^3^ and 3.73 g/cm^3^ for the Al alloy/Ti_2_AlC composites with volume ratios of 40/60 and 27/73, respectively. The volume of the Al alloy is the measured porosity values of Ti_2_AlC foams, assuming all pores were filled with Al alloy. The residual porosity of the composites was measured but was not taken into account for the volume ratio approximation.

The phase composition of the starting powders and the as-infiltrated composites was determined using an X-ray diffractometer, XRD (*D8* Discover, Bruker, WI) with Cu *Kα* radiation (wavelength = 1.542 Å) at 40 kV and 30 mA. The two theta range was from 8° to 80° with a step size of 0.04° and a step time of 1.5 s. The XRD results were analyzed utilizing the Inorganic Crystal Structure Database (ICSD).

The microstructure, phase composition and distribution were characterized using a Field Emission Scanning Electron Microscopy, FE-SEM (JSM-7500F, JEOL, Tokyo, Japan), equipped with Energy Dispersive Spectroscopy (EDS). Also used was another FE-SEM (Zeiss Ultra Plus, Carl Zeiss, Oberkochen, Germany) equipped with an Oxford Instrument AZtec EDS and a Nordlys-S Electron Backscatter Diffraction (EBSD) system. The accelerating voltage and emission current were 15 kV and 20 mA, respectively. The duration of spot scan of EDS was 60 seconds per spectrum. The EBSD scans were run with an accelerating voltage of 12 kV, an aperture size of 60 μm only one EBSD scan is shown here and a step size of 0.3 μm. The 3D microstructure showing the phase distribution was obtained using an Xradia MicroXCT-400 Micro-Computed Tomography (Zeiss, Germany). Further composition measurements were carried out using a Cameca SX-100 electron microprobe with a wavelength dispersive spectrometry (WDS) system.

The compressive strength of the samples was measured by using a universal testing machine (MTS810, MTS, MN) at a strain rate of 1.4 × 10^−4^ s^−1^. The strain was measured using a miniature extensometer (MTS810, MTS, MN) attached to the edges of the compressive grips. All samples were cut by electrical discharge machining to dimensions of 3.5 mm × 3.5 mm × 7 mm to achieve parallelism with less than 1% error of thickness.

## Additional Information

**How to cite this article**: Hu, L. *et al*. High-Performance Metal/Carbide Composites with Far-From-Equilibrium Compositions and Controlled Microstructures. *Sci. Rep.* **6**, 35523; doi: 10.1038/srep35523 (2016).

## Supplementary Material

Supplementary Information

## Figures and Tables

**Table 1:** EDS results of spot analyses in Fig. 2b.

Location	EDS Results					EBSD Results
	Concentration(a), at.%			Ti/Al Ratio	Al/O Ratio	Phase
	Ti	Al	O			
1	0	92	0	···	···	Al
2	24	68	0	0.35	···	TiAl_3_
3	47	24	0	1.96	···	Ti_2_AlC
4	0	37	54	···	0.69	Al_2_O_3_
5	31	69	0	0.45	···	TiAl_2_

EBSD results from Fig. 3 are also listed for comparison. ^(a)^Carbon is not shown in the EDS results because of significant quantifying errors for this specific element.
