# Supplementary material for: High-Performance Metal/Carbide Composites with Far-From-Equilibrium Compositions and Controlled Microstructures
Source: Sci Rep. 2016 Oct 18;6:35523. doi: 10.1038/srep35523 (PMC5067586; doi:10.1038/srep35523)
Supplement: Supplementary Information [file srep35523-s1.pdf]

Supplementary Information

High-Performance Metal/Carbide Composites with Far-From-Equilibrium Compositions and Controlled Microstructures

Liangfa Hu[1], Morgan O'Neil[2], Veysel Erturun[3], Rogelio Benitez[2], Gwénaëlle Proust[4], Ibrahim Karaman[1,2], and Miladin Radovic[1,2]

[1] Department of Materials Science and Engineering, Texas A&M University, College Station, TX 77843, USA. [2] Department of Mechanical Engineering, Texas A&M University, College Station, TX 77843, USA. [3] Department of Airframes and Powerplants, Erciyes University, Kayseri 38039, Turkey. [4] School of Civil Engineering, University of Sydney, NSW 2006, Australia. M.O., V.E., and R.B. contributed equally to this work. Correspondence and requests for materials should be addressed to I.K. (email: ikaraman@tamu.edu) or to M.R. (email: mradovic@tamu.edu).

Correlation of Porosity and Compressive Properties

To illustrate the difference of porosities in these three composites and to relate the porosity difference to the compressive mechanical behavior, **Figure 4**d shows the amount of porosity (both open and closed) in the composites. Note that, whereas the composites with the sizes of the Al alloy phase in the ranges of 167–545 μm and 77–276 μm have similar open and closed porosities, the composites with the sizes of the Al alloy phase in the range of 42–83 μm have significantly lower open and closed porosities than the other two composites. It is well known that porosity of the composites plays an important role in its rupture and thus affect the ultimate compressive strength which decreases significantly with increasing porosity[34]. Thus, the composites with the sizes of the Al alloy phase in the range of 42–83 μm demonstrate the highest compressive strength. It follows from the above that it might be possible to fabricate composites with even smaller sizes of the Al alloy phase and thus higher strengths.

Micro-Computed Tomography Showing the Interpenetrating Network

In order to investigate the three-dimensional (3-D) structure of the processed composites, a series of micro-CT scans were carried out. **Figure S1** shows the micro-CT images of the constituents in the Al alloy/Ti_2AlC composites with sizes of the Al alloy phase ranging from 167 to 545 μm. Unlike the 2-D morphology shown in **Figure 1**, where Al alloy appears to be mostly scattered in individual islands surrounded by Ti_2AlC, the 3-D tomography image shows good connectivity of the Al alloy constituent (**Figure S1**). In fact, both Al alloy and Ti_2AlC form 3-D networks that interpenetrate each other. Note that narrow channels between large pores (more than 100 μm) indicated by arrows in **Figure 2** were also infiltrated with the Al alloy. This suggests that the pore morphology in the MAX phase foams determines the morphology of the infiltrated metallic phase in the composites.

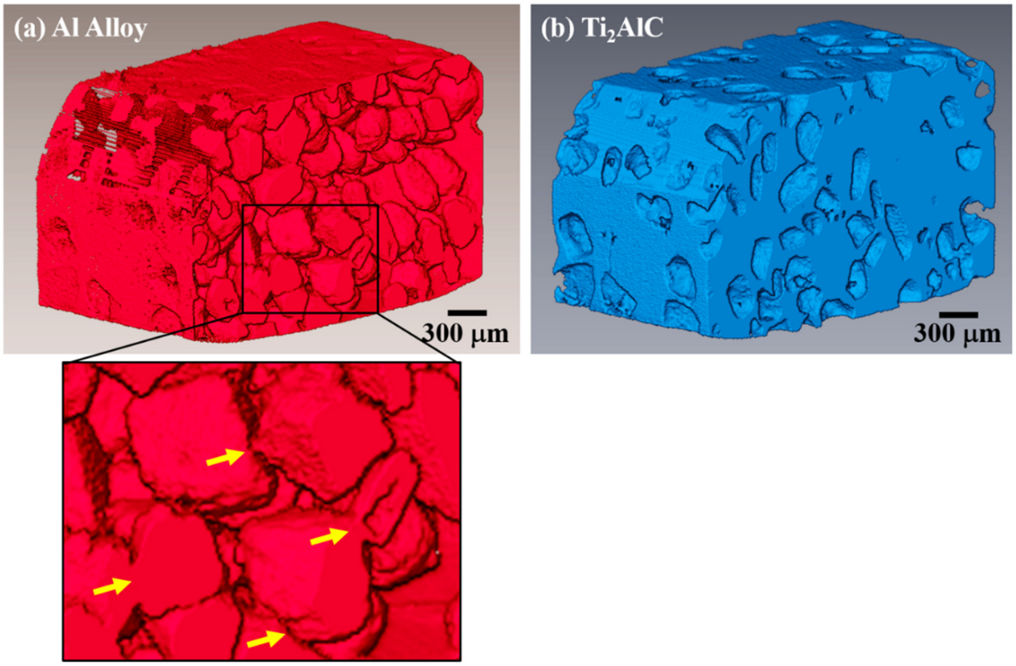

Figure S1. Micro-CT images of the constituents in a cuboid specimen of the composite with Al alloy/Ti_2AlC volume ratio 40/60: (a) Al alloy, and (b) Ti_2AlC phases. The dimensions of the cuboid specimen were 1.8 mm × 1.8 mm × 2.3 mm, and the size of the Al alloy phase is 167–545 μm. Arrows in an the enlarged view of (a) show that narrow channels between large pores (more than 100 μm) were also infiltrated with Al alloy.

Trace Elements of Al Alloy

Al 6061 alloy is a heat-treatable alloy, and the trace alloying elements influence its strengthening. **Figure S2** displays a comparison of the amount of trace elements in the alloy in the composites together with those in a solution heat treated (SHT) Al 6061 alloy. The atomic percent of silicon (Si) and copper (Cu) in the Al alloy significantly decreased after the infiltration process, indicating diffusion of these two trace elements out of the alloy. The diffusion of Cu is most probably driven by reactions between Cu and the ternary carbide and its companion phases [33]. The evidences of Si diffusion are observed in an X-ray map taken around the interfaces inside the composite (**Figure S2c**), and **Figure S2b** shows a back scattered SEM image on the location where the X-ray map was taken. Electron microprobe quantitative analyses show the composition at two locations marked in the back scattered SEM image and the EDS maps (**Figure S2**). The Si concentration is higher at location 1 than at location 2, because location 1 is closer to the Al alloy, and thus to the Si source. In addition, the amount of Si in the Ti_2AlC grains displays a clear, continuous gradient of concentration in **Figure S2c**, with its concentration being high at regions adjacent to the Al alloy but low at regions towards the center of Ti_2AlC grains. The Ti / (Al+Si) ratio approximately equals to 2 at both locations, indicating substitution of Al with Si. The substitution could lead to the formation of $Ti_2(Al_x,Si_{1-x})C$ solid solution or co-existence of two phases, namely Ti_2AlC and Ti_2SiC, which are hardly distinguishable by EDS or EBSD. A further characterization on nanometer scale is required to identify the crystal structure at these two locations.

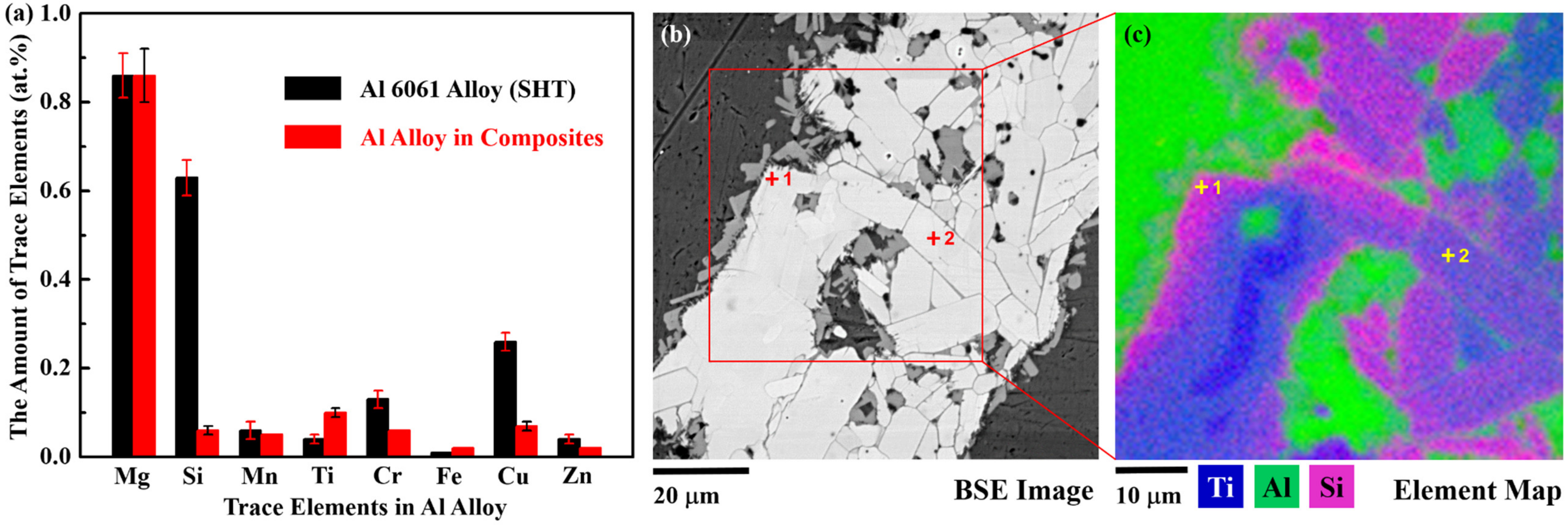

Location	Atomic percent, %				Ti / (Al+Si)	Si / (Al+Si)	Probable phase
	Al	Si	Ti	C			
**1**	20.0	7.3	54.0	18.7	2.0	0.3	$Ti_2(Al_{0.7},Si_{0.3})C$
**2**	25.2	3.1	53.6	18.1	1.9	0.1	$Ti_2(Al_{0.9},Si_{0.1})C$

Figure S2. (a) The amount of the trace elements (in at.%) in solution heat treated (SHT) Al 6061 alloy and the Al alloy in the composites. (b) A back scattered SEM image showing phase distribution (dark: Al alloy, grey: $TiAl_3$, and light: Ti_2AlC) around the Al alloy-Ti_2AlC interfaces inside the composites. (c) An X-ray map showing Si diffusion from the Al alloy to the Ti_2AlC. Red crosses in (b) demonstrate the locations where the electron microprobe quantitative analyses were carried out. The quantitative analyses in spots 1 and 2 on (c) are shown in the table.

Reaction Mechanism

Two possible mechanisms could explain the formation of $TiAl_3$. First, the $TiAl_2$ that were present in the Ti_2AlC powders used to prepare ceramic foams can react with Al to form $TiAl_3$ according to the following reaction:

$$TiAl_2 + Al = TiAl_3 \quad (1)$$

where $TiAl_2$ is an impurity that comes with the commercial MAXthal211 powder. Although the $TiAl_2$ is not shown on the XRD pattern of the Ti_2AlC foam, it is present and identified by EDS in the backscattered SEM images of both Ti_2AlC powder and foams (**Figure 2c**). From SEM images (four randomly selected locations) of Ti_2AlC foams, we measured the area of dark, grey, and light color areas to quantitatively approximate the area percent of Al_2O_3 or pore, $TiAl_2$, and Ti_2AlC or Ti_3AlC_2, respectively. The measurement was determined by using an image analysis software, ImageJ (US National Institutes of Health, Maryland). Similarly, in the EBSD image of the composites, the area percent of $TiAl_3$ was determined (**Figure 3**). The area percent of the $TiAl_2$ in the Ti_2AlC foams of approximately 7 vol.% is equal to that of the $TiAl_3$ in the composites. This is a compelling evidence that the majority of $TiAl_3$ is formed through the reaction given in **Equation 1**.

The other possible mechanism for the formation of $TiAl_3$ is the reaction between Al and Ti_3AlC_2, a common companion phase that comes with the Ti_2AlC powder. In the process of fabricating Ti_3AlC_2/Al composites, Wang et al.[7] found that possible reactions between Al and Ti_3AlC_2 depends on temperature. For temperatures below 900 ^{o}C, the Ti-Al-C ternary phase diagram[29] suggests a reaction path as follows:

$$32\ Al + 3\ Ti_3AlC_2 = 2\ Al_4C_3 + 9\ TiAl_3 \quad (2)$$

Although the presence of $TiAl_3$ was confirmed by both EDS (**Figure 2**) and EBSD (**Figure 3**), the Al_4C_3 (from **Equation 2)** was absent in both cases. The absence of Al_4C_3 could be due to its dissolution by contact with water during polishing according to the following reaction[30]:

$$Al_4C_3 + 12\ H_2O \rightarrow 4\ Al(OH)_3 + 3\ CH_4 \qquad (3)$$

Indeed, both $TiAl_3$ and Al_4C_3 were observed in reactivity studies in the Al-Ti_3SiC_2 system[31,32]. Two observations from the phase map in **Figure 3** enhance the plausibility of the reaction given in **Equation 3**. First, $TiAl_3$ in the ceramic phase region is located close to black dots (unindexed phase) or contains black dots itself. The unindexed, black phases most likely are pores that resulted from the dissolution of Al_4C_3. Second, the $TiAl_3$ "ring" that surrounds the Al phase and separates it from the ceramic phases is non-continuous containing a lot of micron-sized black dots, which are probably pores that resulted from the dissolution of Al_4C_3. Therefore, it is reasonable to assume that reaction proposed in **Equation 2** also takes during infiltration of Al alloy into Ti_2AlC foam.

Inverse Pole Figure Showing the Local Misorientation in a Single Al Alloy Grain

A typical orientation or inverse pole figure (IPF) map of grains in the Al alloy/Ti_2AlC composites is shown in **Figure S3**. Grains with uniform colors denote the ones in which little evidence for plastic deformation was observed. The rainbow colored grains, on the other hand, indicate the ones in which plastic deformation occurred. A zoom-in image of the region within the rectangular frame in **Figure S3a** is shown in **Figure S3b**; the region is labeled as area A. **Figure S3c** shows the trace of the misorientation angle along line B in **Figure S3b**, suggesting that significant deformation occurred in Al alloy during the fabrication process. The misorientation angle between two points, C and D, along the line B within the same Al grain is approximately 12 degrees. These two points are near the two ends of the line and are

approximately 60 μm apart from each other. Some Ti_2AlC grains also showed permanent deformations. Although deformation was observed in only a few Ti_2AlC grains, it is common in most Al alloy grains. The presence of local misorientation and therefore local plastic strains in Al alloy plays an important role in the mechanical response of the composites that will be discussed below.

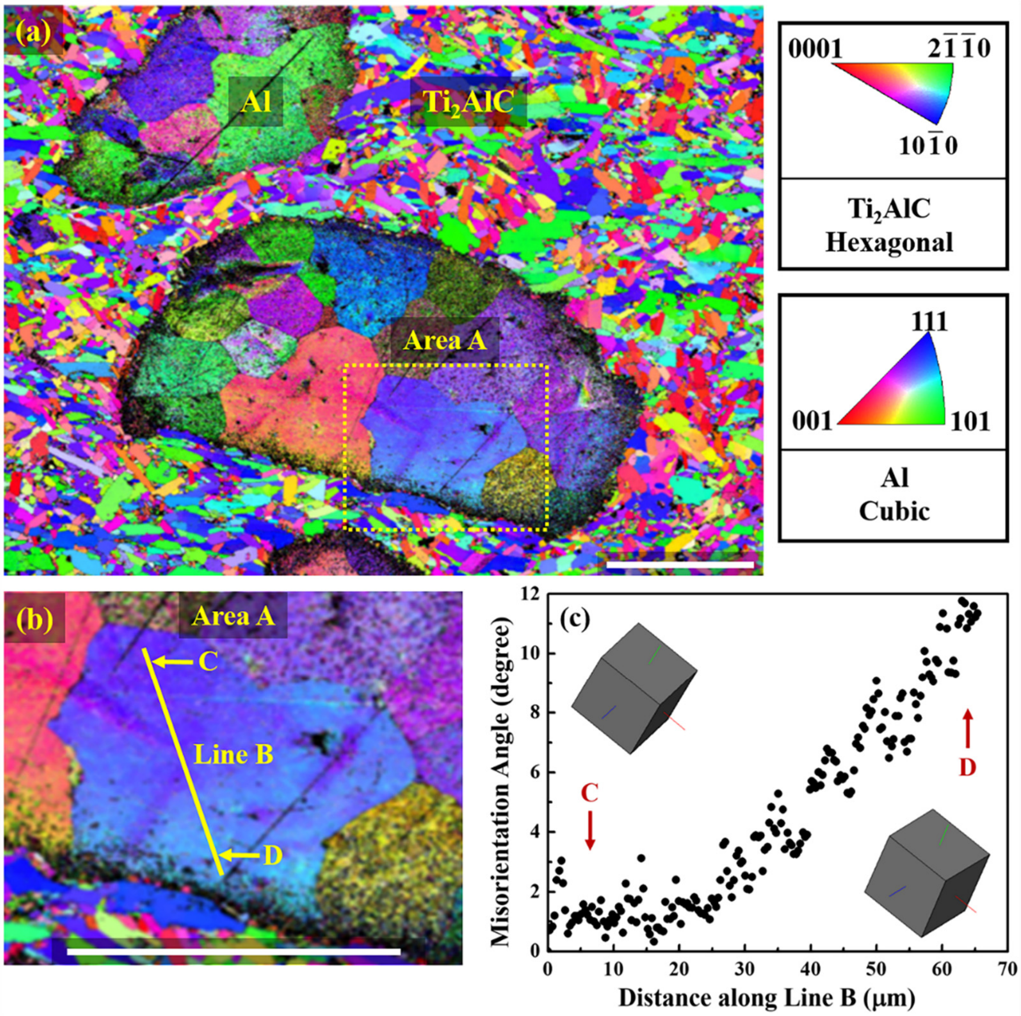

Figure S3. (a) A typical inverse pole figure (IPF) map of grains in the as-processed Al alloy/ Ti_2AlC (volume ratio 40/60) composites. Grains with uniform colors denote the ones in which little evidence for plastic deformation was observed (which is mostly the case for the ceramic grains). The rainbow colored grains (which are almost all the Al alloy grains), on the other hand, indicate the ones in which plastic deformation occurred and has resulted in local misorientations. (b) A zoom-in image of the region within the rectangular frame in (a); the region is labeled as area A. (c) trace of the misorientation angles along the line B in (b), indicating that significant deformation occurred in the Al alloy grain.

Mechanical Properties

The presence of the reaction phase ($TiAl_3$) in the composites, despite of the small amount, also may play a role in the compressive properties. First, the $TiAl_3$ located inside the pores in the ceramic walls filled molten Al and its presence could reduce the exposure of the composites to crack initiations from small pores. Second, the $TiAl_3$ located in the interfacial area increases the density of the phase boundaries and could provide an additional resistance to crack propagation. Third, after the rapid cooling, the coexistence of the intermetallic ($TiAl_3$) and the MAX phase could introduce residual stresses in the composites. Last but not least, the difference in elastic moduli of the intermetallic ($TiAl_3$) and the MAX phase could lead to uneven load partitioning during loading. The last two points were discussed in another study for a similar intermetallic-carbide system (NiTi-Ti_3SiC_2)[9]. The exact contribution of each effect above is quite difficult to distinguish. However, it is believed that the last two contributions are not significant as compared to the contributions from the porosity and the ceramic-metal interfaces due to the low volume percent of $TiAl_3$.

The trade-off between strength and ductility is not unusual and has been observed in other Al-ceramic composites. Compressive properties, *i.e.* yield strength, ultimate strength, and failure strain, at both room and elevated temperatures of a number of Al-ceramic composites are listed in **Table S1**. These composites include the Al alloy/Ti_2AlC from the present study, Ti_3AlC_2/Al[7], Al_2O_3/Al-2wt.%Cu[14], B_4C/Al[17], Al_2O_3/Al-13Si-9Mg[17], and Al_2O_3/Al[15]. According to the data in **Table S1**, the specific strength as a function of compression test temperature for Ti_3AlC_2/Al[7], B_4C/Al[17], and Al_2O_3/Al[15] composites was plotted in **Figure 5d** for comparison with that for the Al alloy/Ti_2AlC composites examined in this study and Al 6061 alloy. The specific strength of the Al alloy/Ti_2AlC composites is the highest among other Al-ceramic composites

previously reported in the literature. Note that the Al alloy/Ti_2AlC composites in the present study were processed by infiltration of molten Al alloy in pre-sintered Ti_2AlC foams, whereas other composites were processed by co-sintering of Al-ceramic powder mixtures. The difference in compressive strength between the Al alloy/Ti_2AlC composites and other composites could be related to constituent materials and the microstructural differences originated from the different processing methods. The remainder of this section demonstrates these differences and their correlation to compressive properties. The Ti_3AlC_2/Al composites[7] were chosen to represent the Al-ceramic composites processed by conventional methods.

Table S1. Compressive properties (*i.e.* yield strength, ultimate strength, and failure strain) at both room and elevated temperatures of various Al-ceramic composites.

Composite systems		Volume percent of ceramic (%)	Properties at room temperature			Properties at elevated temperatures				Reference
Aluminum	Ceramic		Yield strength (MPa)	Ultimate strength (MPa)	Failure strain (%)	Yield strength (MPa)	Ultimate strength (MPa)	Failur e strain (%)	Test temperature (^oC)	
6061 alloy	Ti_2AlC	73	1035±20	1095±20	1	755	800	1	400	This work
6061 alloy	Ti_2AlC	60	630±30	668±28	1	400±30	426±37	1	400	This work
Al	Ti_3AlC_2	40	174	190	4	145	160	2	175	[7]
						100	110	1	325	
						65	80	1	475	
Al-2wt.%Cu	Al_2O_3	30	400	520	5	…	…	…	…	[14]
Al	B_4C	55	350	400	4	100	140	10	375	[17]
Al-13Si-9Mg	SiC	48	…	405	…	…	…	…	…	[20]
Al	Al_2O_3	34	390	464	3	236	284	3	250	[15]
						85	87	1	500	

Microstructural Evidence of Crack Propagation Resistance

Figure S4 shows SEM images of the post-compression composites presenting crack deflection before the metallic phase and crack arresting and bridging by the metallic phase. As shown by the photograph in **Figure S4a**, all tested samples failed by cracking in the direction of approximately 45 ^{o} relative to the loading direction with a tortuous crack path. This observation suggests that the composite shows a graceful failure and does not crush and fragment under compression. Higher magnification SEM images in **Figure S4** of the fracture surface area clearly show that cracks in the ceramic phase were effectively arrested (**Figures S4c–e**), deflected (**Figures S4b and S4c**), or bridged (**Figure S4b, S4d, and S4f**) by the metallic phase. More importantly, no crack between Ti_2AlC and Al alloy has been observed after compression testing, indicating exceptionally strong bonding between the two phases in the composite.

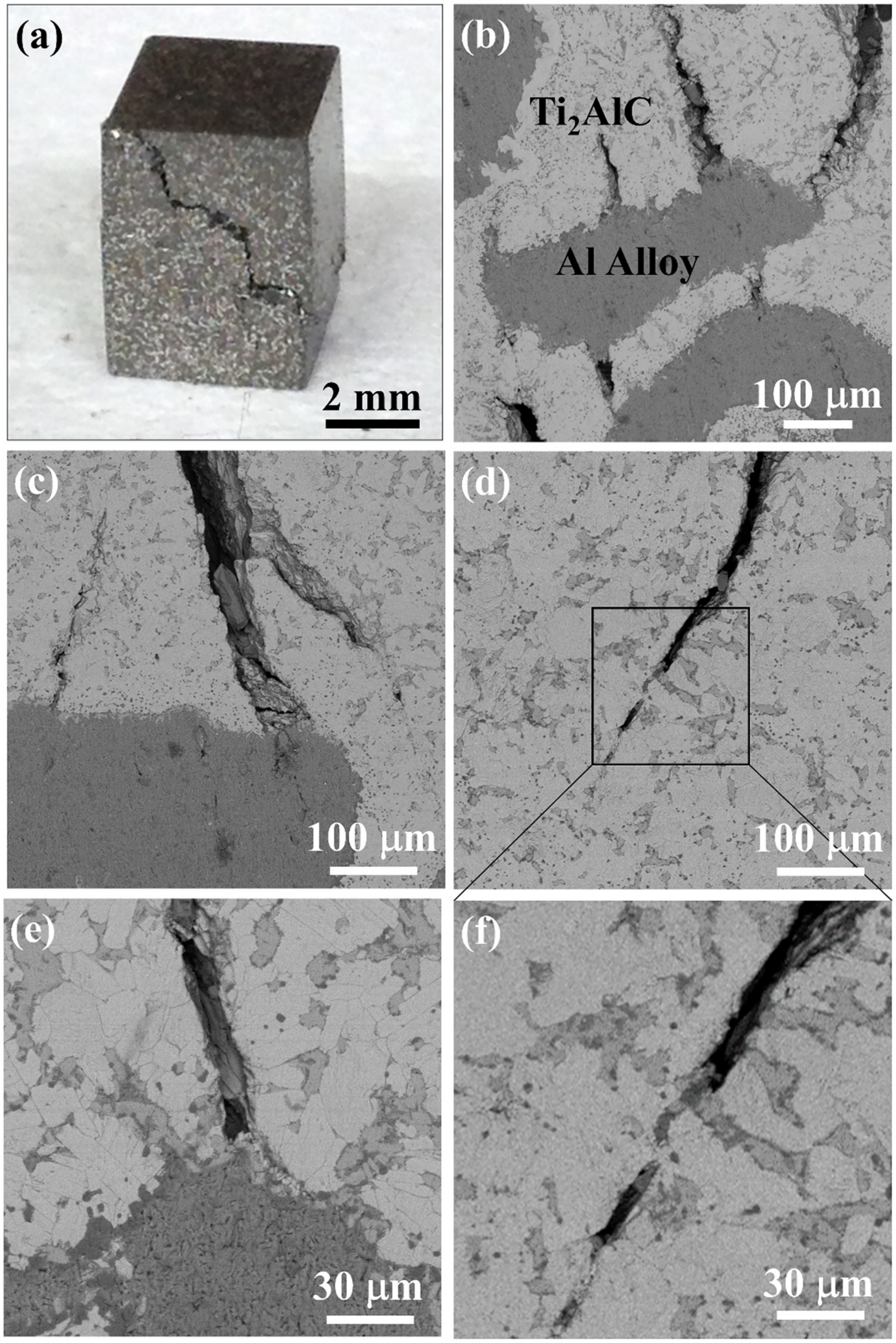

Figure S4. Surface morphology of a post-compression Al alloy/Ti_2AlC (volume ratio 40/60) composite. Loading was in the vertical direction. The sample failed by cracking in the direction of approximately 45 ^{o} relative to the loading direction with a tortuous crack path. The SEM images indicate that cracks in the ceramic phase were arrested ((b), (c), and (e)), deflected ((b) and (c)), or bridged ((d) and (f)) by the metallic phase.
